# Supplementary material for: Factors motivating maternal healthcare clients to use mHealth interventions in rural Malawi
Source: PLOS Digit Health. 2025 Apr 22;4(4):e0000805. doi: 10.1371/journal.pdig.0000805 (PMC12013878; doi:10.1371/journal.pdig.0000805)
Supplement: S1 Text — (DOCX) [file pdig.0000805.s003.docx]

The questions below will be used as a guide for the interviews with maternal clients who do not own a mobile phone. The question may be rephrased and probed in various ways.

**Section A: Demographic Information**

1. What is your age range?
2. 15-19
3. 20-25
4. 26-35
5. 35-45
6. What is your level of education?
7. Primary School
8. Junior Secondary School
9. Senior Secondary School
10. Tertiary education
11. None
12. How many pregnancies have you used this intervention?
13. Who is the owner of the mobile phone you use for CCPF?
14. If you use a borrowed mobile phone, what motivated you to use borrowed mobile phones to access the intervention?
15. Did the owner of the mobile phone persuaded you to use the intervention?

## Section B: Extrinsic Motivation

1. When did you enrol for the intervention?
2. How did you learn about the Chipatala Cha Pa Foni?
3. Describe the reasons why you enrolled for the intervention
4. May you describe how you enrolled into the intervention
5. Who did you discuss with about the intervention before decision to enrol? Why?
6. What motivated you to use the intervention?

**Section C: Basic psychological needs**

1. Describe how you use / engage with the intervention.
2. How did other people help you to use the intervention?
3. What challenges do you face when using the intervention?
4. What technical aspects of the intervention can you talk about? You can talk about the mobile phone, CCPF system etc.

## Section D: Intrinsic motivation

1. After using the intervention, what do you think motivated you to use the intervention?
2. What are your views about the mHealth intervention?
