# Supplementary material for: Factors motivating maternal healthcare clients to use mHealth interventions in rural Malawi
Source: PLOS Digit Health. 2025 Apr 22;4(4):e0000805. doi: 10.1371/journal.pdig.0000805 (PMC12013878; doi:10.1371/journal.pdig.0000805)
Supplement: Attachment — Submitted filename: PDIG-D-24-00236 _ Editor comments.pdf [file pdig.0000805.s004.pdf]

PLOS Digital Health

Factors motivating maternal healthcare clients to use mHealth interventions in rural Malawi

--Manuscript Draft--

Manuscript Number:	PDIG-D-24-00236
Article Type:	Research Article
Full Title:	Factors motivating maternal healthcare clients to use mHealth interventions in rural Malawi
Short Title:	Motivating factors for mHealth use in rural Malawi
Corresponding Author:	Priscilla Maliwichi, Msc., BSc<br>Malawi University of Science and Technology<br>Thyolo, MALAWI
Order of Authors:	Priscilla Maliwichi, Msc., BSc
	Wallace Chigona, PhD, MSc, BSoc
	Address Malata, PhD, MSc, BSc
Keywords:	maternal healthcare; mHealth interventions; self-determination theory; motivation; enablers of mHealth
Abstract:	Client-facing mHealth interventions have the potential to address the inequalities in accessing health information. In maternal healthcare, mHealth interventions provide information to pregnant women on how they can stay healthy during pregnancy, as well as on the danger signs in pregnancy that can contribute to maternal mortality. Sub-Saharan Africa has the highest maternal deaths in the world, and most of these deaths are preventable. In this study, we employed qualitative research methods to investigate factors that motivate maternal healthcare clients in rural Malawi to use mHealth interventions. We used a conceptual framework informed by self-determination theory as the theoretical lens for the study. Data was collected using secondary data sources and semi-structured interviews with maternal clients who used the mHealth intervention. The study found enablers to mHealth use such as ownership or psychological ownership of mobile phones and persuasion to use mHealth intervention motivated maternal healthcare clients to use the mHealth interventions. Furthermore, women in rural areas might be motivated to use mHealth interventions when the technology suppresses social-cultural norms, technology is designed with affordance potency in mind, women have trust in the source of information, and when communities practice the culture of sharing. These findings have the potential to broaden the understanding of what and why beneficiaries of digital health might be motivated to use digital technologies in poor-resource settings. The study may also inform mHealth designers and implementers on how to design and implement mHealth interventions in poor resource settings.
Suggested Reviewers:	
Opposed Reviewers:	
Additional Information:	
Question	Response
**Government Employee**<br><br>Are you or any of the contributing authors an employee of the United States government?<br><br>Manuscripts authored by one or more US	No - No authors are employees of the U.S. government.

Powered by Editorial Manager® and ProduXion Manager® from Aries Systems Corporation

Government employees are not copyrighted, but are licensed under a CC0 Public Domain Dedication, which allows unlimited distribution and reuse of the article for any lawful purpose. This is a legal requirement for US Government employees.<br><br>This will be typeset if the manuscript is accepted for publication.	
**Financial Disclosure**<br><br>Enter a financial disclosure statement that describes the sources of funding for the work included in this submission. Review the submission guidelines for detailed requirements. View published research articles from *PLOS Digital Health* for specific examples.<br><br>This statement is required for submission and **will appear in the published article** if the submission is accepted. Please make sure it is accurate.<br><br>**Funded studies**<br>Enter a statement with the following details:<br>• Initials of the authors who received each award<br>• Grant numbers awarded to each author<br>• The full name of each funder<br>• URL of each funder website<br>• Did the sponsors or funders play any role in the study design, data collection and analysis, decision to publish, or preparation of the manuscript?<br><br>Did you receive funding for this work?	The author(s) received no specific funding for this work.
**Competing Interests**<br><br>On behalf of all authors, disclose any competing interests that could be perceived to bias this work.<br><br>This statement will be typeset if the	None

manuscript is accepted for publication.<br><br>Review the instructions link below and PLOS Digital Health's competing interests policy to determine what information must be disclosed at submission.	
**Data Availability**<br><br>Provide a **Data Availability Statement** in the box below. This statement should detail where the data used in this submission can be accessed. This statement will be typeset if the manuscript is accepted for publication.<br><br>Before publication, authors are required to make all data underlying their findings fully available, without restriction. Review our PLOS Data Policy page for detailed information on this policy. Instructions for writing your Data Availability statement can be accessed via the Instructions link below.	Data will be available after the thesis has been uploaded under UCT Library using UCT ZivaHub - open data UCT at the end of the project.

Powered by Editorial Manager® and ProduXion Manager® from Aries Systems Corporation

Cover Letter

Dear Editor,

This paper looks at factors motivating maternal healthcare clients in poor resource settings to use maternal mHealth interventions. The study found enablers to mHealth use such as ownership or psychological ownership of mobile phones and persuasion to use mHealth intervention motivated maternal healthcare clients to use the mHealth interventions. Furthermore, women in rural areas might be motivated to use mHealth interventions when the technology suppresses social-cultural norms, technology is designed with affordance potency in mind, women have trust in the source of information, and when communities practice the culture of sharing. These findings have the potential to broaden the understanding of what and why beneficiaries of digital health might be motivated to use digital technologies in poor-resource settings.

The paper contributes to the understanding of Self-determination Theory and the study conceptualised enables to use mHealth. In poor resource setting owning mobile phones enables clients to access an mHealth intervention. In an event where a beneficiary of mHealth does not own a prerequisite technology, psychological ownership of the mobile phone of a third party enables someone to have access to an intervention. Additionally, in these settings, clients need to be persuaded to use interventions. Therefore, this study looks into e perspective of users of mHealth who own and those who do own mobile phones. Most studies in mHealth only concentrate on those who own technologies such as mobile phones. Hence, the study may also inform mHealth designers and implementers on how to design and implement mHealth interventions in poor resource settings.

The manuscript is a research article that has never been submitted to PLOS digital health before. The appropriate Academic Editor could be someone who is into digital health such Gianmarco Abbadessa and Mohsen Abbasi-Kangevari. At the moment I do not have any opposed reviewers.

Yours sincerely,

Priscilla Maliwichi

Manuscript Click here to access/download;Manuscript;Manuscript PM.pdf

Factors motivating maternal healthcare clients to use mHealth interventions in rural Malawi

Priscilla Maliwichi [1,2,*], Wallace Chigona [1], Address Malata [3]

[1] Department of Information Systems, University of Cape Town, Cape Town, Western Cape, South Africa

[2] Department of Computer Science and Information Technology, Malawi University of Science and Technology, Thyolo, Southern Region, Malawi

[3] Administration Department, Malawi University of Science and Technology, Thyolo, Southern Region, Malawi

[*] Corresponding author

Email: pmaliwichi@must.ac.mw

// Abstract

Client-facing mHealth interventions have the potential to address the inequalities in accessing health information. In maternal healthcare, mHealth interventions provide information to pregnant women on how they can stay healthy during pregnancy, as well as on the danger signs in pregnancy that can contribute to maternal mortality. Sub-Saharan Africa has the highest maternal deaths in the world, and most of these deaths are preventable. In this study, we employed qualitative research methods to investigate factors that motivate maternal healthcare clients in rural Malawi to use mHealth interventions. We used a conceptual framework informed by self-determination theory as the theoretical lens for the study. Data was collected using secondary data sources and semi-structured interviews with maternal clients who used the mHealth intervention. The study found enablers to mHealth use such as ownership or psychological ownership of mobile phones and persuasion to use mHealth intervention motivated maternal healthcare clients to use the mHealth interventions. Furthermore, women in rural areas might be motivated to use mHealth interventions when the technology suppresses social-cultural norms, technology is designed with affordance potency in mind, women have trust in the source of information, and when communities practice the culture of sharing. These findings have the potential to broaden the understanding of what and why beneficiaries of digital health might be motivated to use digital technologies in poor-resource settings. The study may also inform mHealth designers and implementers on how to design and implement mHealth interventions in poor resource settings.

Introduction

Use of health facilities for pregnancy-related issues improves maternal outcomes. However, a lack of infrastructure such as health facilities in rural areas of African countries is a concern in maternal healthcare (1). In most cases, maternal healthcare clients in rural areas travel long distances to access maternal healthcare services, such as antenatal care, delivery, and postnatal care (2). The long distances contribute to poor maternal outcomes (3). Many governments and development agencies implement digital health such as mobile technology health (mHealth) interventions to reduce the maternal mortality ratio (MMR) to meet the Sustainable Development Goal (SDG) 3.1 (2,4). SGD 3.1 seeks to reduce global maternal mortality to below 70 deaths per 100,000 births (1). mHealth interventions have the potential to improve health-seeking behaviour, thereby increasing health facility usage.

Maternal mHealth interventions in rural areas of African countries are bedeviled by a myriad of challenges *inter-alia* low mobile phone ownership, especially amongst women (5). Malawi, like other low-income countries, has a low penetration rate of mobile phones; currently standing at 45% (6). The mobile penetration and mobile phone ownership is even lower in rural areas (7). Women in rural areas of Sub-Saharan Africa are 15% less likely to own a mobile phone than their male counterparts (8). It is therefore understandable that the adoption and use of maternal mHealth intervention is low (9).

which m-health intervention? or do you mean generally maternal m-health intervention uptake?

About 27% of the population where the maternal mHealth intervention was piloted in Malawi adopted and used the intervention (9). In that context, users of the mHealth intervention had to surmount several challenges to use the intervention. For example, some women who do not own a mobile phone used a shared mobile phones to access the interventions (2). Despite these challenges, the maternal healthcare clients used maternal mHealth interventions. It is, therefore, important to understand the factors that motivated rural women to use maternal mHealth intervention.

Provide further detail here - what % of women used it? is any usage data available? Are you talking about a specific intervention? Provide some context about the study here.

Motivation is defined as that drive which pushes someone to do things in order to achieve a target (10). Most studies in motivation within the mHealth space have concentrated on motivating factors influencing health workers to use mHealth (11,12). Furthermore, studies in motivation that concentrated on healthcare client-side occurred in developed countries where penetration of mobile phones is high (13,14). However, there is a dearth of literature on motivating factors influencing healthcare beneficiaries to use mHealth interventions in poor resource settings of developing countries (15,16). This study focused on maternal mHealth intervention since maternal health is challenged by social-cultural norms, which may hinder the adoption and use of mHealth interventions. We posit that a range of motivating factors influences maternal healthcare clients to use maternal mHealth interventions. Consequently, the research question of this study is as follows:

- Why are healthcare clients in rural Malawi motivated to use maternal mHealth interventions?

To answer the research question, we used qualitative research methods and self-determination theory (SDT) as a theoretical lens. We used a case study of Chipatala Cha Pa Foni (CCPF) – which translates to Health Centre by Phone. CCPF is a maternal and child health intervention piloted in a poorly performing district in Malawi with regards to maternal and child health. Users of the intervention (maternal healthcare clients) call the toll-free hotline and listen to pre-recorded messages or receive a short message service (SMS) on maternal health. The mHealth intervention caters for maternal healthcare clients who either own or do not own mobile phones (17). Malawi was an appropriate case study, since the country has one of the highest MMR in Southern Africa, at 349 per 100000 live births (NSO, 2019). Further to this, the country has a low mobile adoption rate. The findings of the study may inform designers and implementers of mHealth interventions on what to focus on to motivate potential beneficiaries to adopt and benefit from interventions.

Materials and methods

The study employed a qualitative research methodology. Even though SDT has benefitted from quantitative experimental studies, SDT can be used to test hypotheses even using traditional approaches such as qualitative research methods (18). For example, SDT was applied using a realist approach to enhance the explanatory nature of evaluations (19). SDT was used for this study since it is an explanatory study and seeks to determine why maternal healthcare clients were motivated to use mHealth interventions. A qualitative research methodology was used since it is subjective in the sense that it points to the role of human subjectivity in the research process and provides new meaning to knowledge (20). The study used a single case study. This study examined a group of women within an environment in which there were poor maternal health outcomes (2). We used a purposive sampling method to sample the maternal healthcare clients.

CCPF case study

CCPF was introduced in 2011 as an intervention aimed to decrease maternal and child deaths (Mobile Alliance for Maternal Action, 2013). The main objective of CCPF project was to maximise healthcare access and utilisation by remote maternal healthcare clients facing the many significant challenges, such as walking long distances to access a health facility, resulting in delays in seeking care and unnecessary expenditures.

The project started as a pilot project of the Concern Worldwide innovations for Maternal, Newborn, and Child Health, and was implemented by VillageReach in the Balaka District of Malawi. The Ministry of Health and the project implementers chose Balaka District as a pilot site for the project

from 2011 to 2016. They chose the district due to its poor performance in maternal health. Only 35% of the women with live birth had attended antenatal care during the first trimester, and about 32% of the women delivered at home (22).

Components of the CCPF System

At first, CCPF had two main components: i) toll-free case management hotline (which is available on an Airtel Malawi mobile line); and ii) tips and reminders. These components were designed to work on a basic mobile phone, which is common in poor-resource settings.

Case Management Hotline

The toll-free case management hotline was stationed at the district hospital and was managed by qualified hotline workers (HLWs). The HLWs were trained in maternal and child health community case management; this is a training that is also provided to CHWs. Maternal healthcare clients were told about their expected date of delivery (EDD), the current stage of pregnancy, and HLW could answer any pregnancy-related questions.

1. Tips and Reminders

Tips were personalized messages according to the stage of the pregnancy. Reminders were messages for antenatal appointments, medication, and supplements during pregnancy. The messages were in two vernacular languages widely spoken in the District. The voice messages were retrieved on any Airtel line upon authentication, using the EDD and password (voice messages were common for non-mobile phone owners). Text messages were sent direct to the personal mobile phone.

After the pilot phase, CCPF was scaled-up and handed over to the Government of Malawi in 2018 (23). CCPF is now available in all districts of Malawi around the clock, seven days a week. The tips and reminders component has been replaced with pre-recorded voice messages and everyone can access

them using an Interactive Voice Response (IVR) system when they call the toll-free number. The callers choose whether they want to speak to a hotline worker or listen to the voice messages.

2. Community Volunteers for CCPF

The implementing agency recruited about 400 community volunteers across the four catchment areas and each village was assigned a community volunteer. Community volunteers were people within the community. The minimum qualification for the volunteers was that they should have basic literacy and could use a mobile phone. The volunteers were not Health Surveillance Assistants (HSAs) (in Malawi HSAs are CHWs employed by the government). Their role was to provide maternal healthcare clients with access to a mobile phone for the intervention and demonstrate how to use the system. In addition, community volunteers were visiting maternal healthcare clients in their homes for registration and follow up on tips and reminders so that women could listen to their messages. The project provided volunteers with mobile phones to be used for CCPF for maternal healthcare clients in their communities. Other maternal healthcare clients were using their own mobile phones, other community members or family members' mobile phones. This was the case because CCPF is available for free on one mobile operator (Airtel Malawi), so maternal healthcare clients who have mobile numbers for other mobile operators had to find someone with an Airtel line to use the toll-free number or listen to their messages

Data Collection

Data was collected using secondary data sources on CCPF and semi-structured interviews with maternal healthcare clients in 2019. Secondary data relating to the project were collected from the internet, as well as accessed directly from the project, and peer-review research outputs. For project documents and peer-review articles from the internet, we used Google and Google Scholar databases to search for articles. We used the search terms: maternal health. mHealth, CCPF, Chipatala Cha Pa Foni and Malawi. We used secondary data to develop the context of the study. Project reports provided a good window to the background and the main objectives of the intervention, and

highlighted some activities and processes involved in the maternal healthcare clients' use of the intervention. Figure 2 shows the PRISMA diagramme, which summarises the step used to collect secondary data. ***SI Table 1*** summarises secondary data used in this study.

Fig 2. *PRISMA diagram showing steps used to collect secondary data*

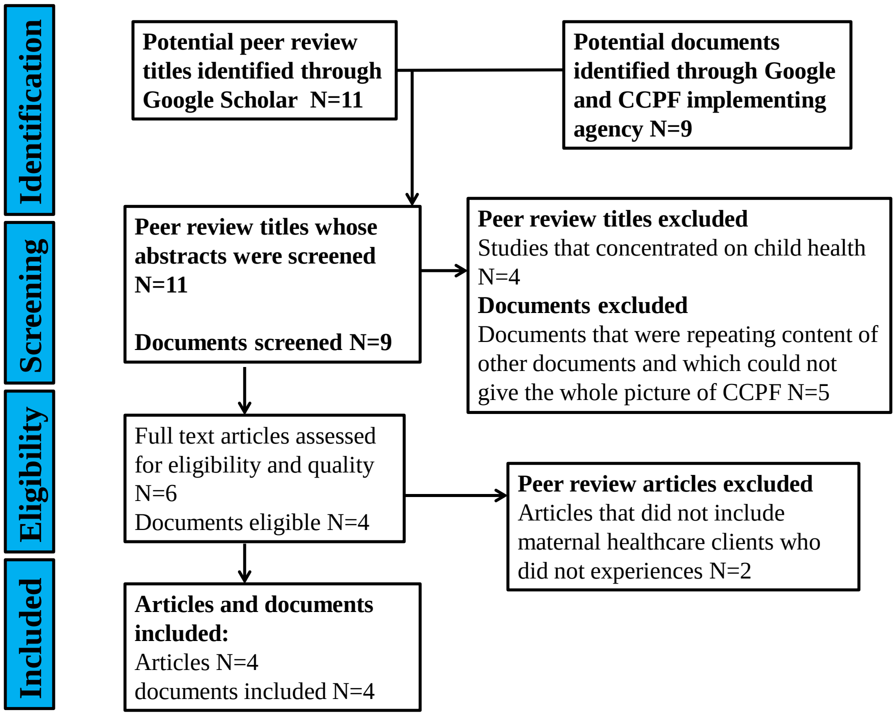

We conducted semi-structured interviews with the maternal healthcare clients. The sample had 20 maternal healthcare clients and the saturation point was reached when we could not get any new data from the participants. All the maternal healthcare clients had attended the minimum required antenatal visits and had all delivered at the health facility. ***SI Table 2*** summarises the demographic characteristics of the maternal healthcare clients.

We used the following procedure to access the respondents:

- The project team queried the Caller Database for CCPF for the period of August 2017 to December 2018 to obtain mobile numbers for maternal healthcare clients who called or registered for CCPF in Balaka District using their own mobile phones or borrowed mobile phones. We chose this period to find more active mobile numbers. We identified “hotspots” (areas which made more calls from the caller’s database) in Balaka District. We were aware of the ethical and privacy issues involved when using mobile numbers from the callers database. Hence, when the call connected to the potential participants, we introduced ourselves as researchers studying CCPF, and we are calling people randomly to know if they know CCPF. If they know about CCPF, we proceeded as described in section 4.3.
- In the case where a maternal healthcare client used a borrowed mobile phone, we asked the mobile phone owner about CCPF, and whether they know anyone who has used their mobile phone for CCPF. Since CCPF was initially launched as a maternal and child health intervention in the District, it was easy for the mobile phone owner to identify the maternal healthcare clients who had used their mobile phone for CCPF. Fortunately, the owners of the mobile phones were husbands of the maternal healthcare clients, community volunteers and female community leaders. This made access to maternal healthcare clients who do not own mobile phones easy. For ethical and privacy issues, we proceeded as described in section 4.3.

The interviews took 20 to 30 minutes each and were conducted in Chichewa. Chichewa is a national local language of Malawi, so all respondents understood the language. For each interview, the researcher took notes and recorded the calls using CallX mobile application.

Please provide interview questions as supplementary materials, and include examples of questions asked in the paper (If possible, please provide English translation of interview questions).

Data Analysis

We conducted data analysis in two phases. The first phase focused on document analysis and the second phase focused on empirical data collected using semi-structured interviews. The audio-recordings were transcribed and coded using qualitative research software (Nvivo 12). We employed a thematic analysis to analyse the data. Several phases, as stipulated by Braun and Clarke (24) guided the analysis process. Table 2 summarises the steps we took in data analysis.

Please unpack the SDT further - what does it say? what are the components of self-determination, etc. according to this theory?

Table 2. Summary of data analysis steps

Phases of thematic data analysis	Activities done
Familiarisation	Translated and transcribed the audio interviews from Chichewa into English. The raw transcribed data (including the notes) was then imported into Nvivo 12 for analysis.
Theme development and coding	Data from secondary sources and empirical data was coded in Nvivo 12. Author 1 used the concepts from SDT and new themes emerging from the data were used to create themes and subthemes (nodes and sub-nodes) in Nvivo 12. Using the whole data set, the researcher finalised coding the data.
Reviewing themes	Author 2 reviewed the coded data extracts, where collated data extract of each theme were read to determine whether the current theme made a coherent pattern. If the data extract did not fit, they were moved to themes where they fit.

	The validity of individual themes in relation to the entire data set was also checked. Using Nvivo 12, both authors developed a thematic diagram (*see Figure 2*) to see if it accurately reflects the whole data set's meaning based on SDT and emerging new themes.
Defining and naming	Defining and refining the themes that are going to be present in the finding chapter. We identified what was of interest in the data set and why, and the story that each theme tells.
Writing up	The findings section of this study present the results and the interpretation of the analysed data.

Ethical considerations

Before data collection, we obtained permission to use CCPF as a case study from the implementing agency of CCPF, Malawi Ministry of Health and Balaka District Health Office. Furthermore, we obtained ethical clearance from the National Health Sciences Research Committee (Malawi).

Please provide ethics approval / project ID if possible

During the interview sessions, the researchers introduced themselves as researchers studying CCPF Project. Consent was sought before interviews started and issues of privacy and confidentiality of the data collected were discussed. For maternal healthcare clients who did not own mobile phones, we had to seek consent to interview them via the mobile phone owners before they could come forward

Was written consent sought? Or verbal? If verbal, was consent recorded? Also what are the challenges of informed consent? Did your participants have adequate literacy levels to understand the project? how did you ensure that the consent they were giving was informed consent?

for the interviews. We were aware of the risks of interviewing pregnant women: there could be an

Women who are no longer pregnant but had difficulties during pregnancy or childbirth could also recall such memories…

emergency during interviews, or women could recall traumatic experiences related to the pregnancy. To mitigate against such risks, our sample was limited to mothers who were not pregnant at the time of data collection; they were all women who had previously used the system. Further to this, we informed the respondents that participation of the study was voluntary, and they could withdraw from the study any time. For the analysis, we anonymized the maternal healthcare clients as Client x.

Findings

The findings of this study suggest that several factors motivated maternal healthcare clients to use the maternal mHealth intervention. Based on SDT we categorised the results as extrinsic motivating factors, basic psychological needs, internalization and integration and intrinsic motivating factors. Furthermore, we had emerging theme which we conceptualized as enablers to use mHealth interventions. This was conceptualized further into ownership or psychological ownership of mobile phones and persuasion to use mHealth intervention. Figure 2 shows all the themes derived from the study.

Fig 2. Motivating factors, enablers, and basic psychological needs that motivated maternal healthcare clients to use mHealth interventions based on SDT

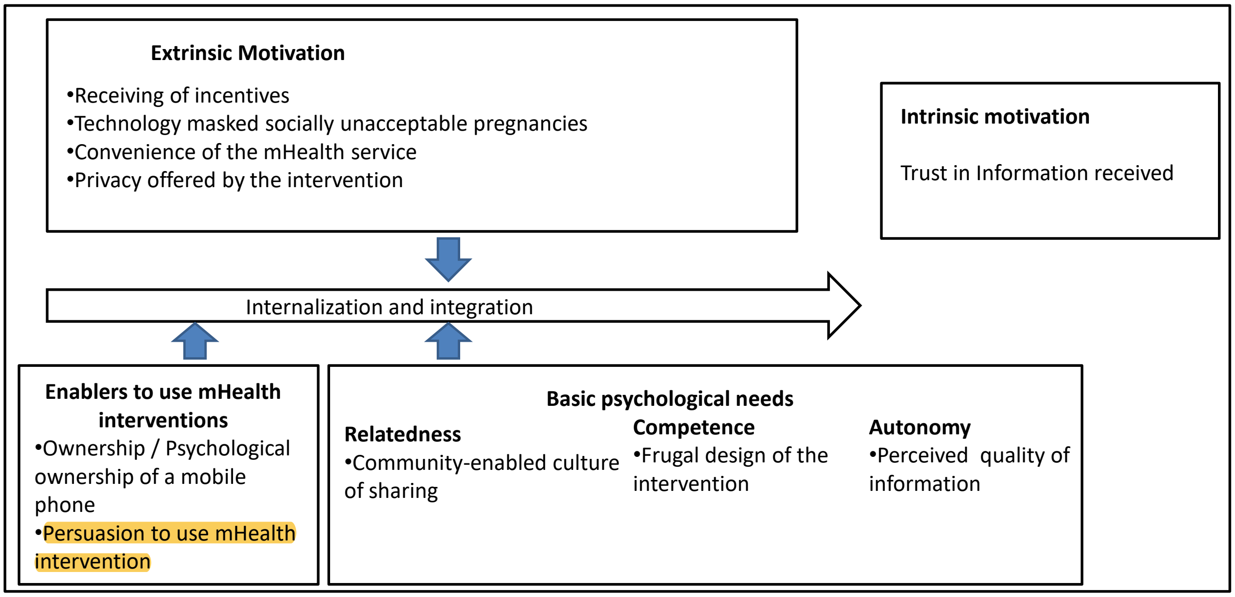

unsure what you mean by this specifically …

Extrinsic motivating factors

Several extrinsic factors were identified as motivating maternal healthcare clients to use maternal mHealth interventions and these were: receiving of incentives; technology masked socially unacceptable pregnancies; convenience of the mHealth service; and privacy offered by the intervention.

Receiving of incentives

During the pilot phase of CCPF, maternal healthcare clients were encouraged to register for CCPF because the project implementers were giving out mosquito nets to maternal healthcare clients, who registered for CCPF during pregnancy. However, after the pilot phase, CCPF project implementers scaled-up CCPF to other districts, and mosquito nets were no longer being distributed in the Balaka District. However, maternal healthcare clients were expecting to be given mosquito nets as a compensation for using CCPF mHealth intervention.

"In the early days of the project, we used to receive mosquito nets when we registered for CCPF. However, we no longer receive the mosquito nets …" [Client 6].

realising

This finding shows that the maternal healthcare clients might be disappointed after releasing that the mosquito net were no longer available. However, despite the incentive being removed at the clinics in Balaka, maternal healthcare clients continued to use CCPF, because they saw its importance.

Technology masked socially unacceptable pregnancies

suggest different wording - outside of a marriage

The maternal healthcare clients in this study were motivated to use the mHealth intervention since no one would judge their pregnancy. This finding was common among unmarried pregnant women, as well as teenage girls who were pregnant out-of-wedlock. It could be the case that in a rural setting, society does not accept the pregnancy of unmarried women or teenage girls. However, the mHealth intervention provided teenage healthcare clients an avenue where they could get information about pregnancy and what they are supposed to do during pregnancy, without judgment.

"I was shy to go to the hospital to ask certain things when I was pregnant because I was not yet married, so when I heard of this toll-free number that we can call and ask questions, I decided to register" [Client 2].

what are the other reasons for this? Is it just the above that you have described?

mHealth intervention has the potential to unlock access to maternal healthcare information to maternal healthcare clients whose pregnancies are socially unacceptable. For example, in settings where religious as well as cultural traditions are important, pregnant women who could not conform to these religious or cultural and tradition norms could be potentially excluded from accessing proper maternal healthcare services due to shame.

Convenience of the mHealth service

The maternal healthcare clients in this study could use the hotline of CCPF at anytime of the day since CCPF is available around the clock. Maternal healthcare clients could call the hotline anytime while at home. *"It is a quick hospital service, they help us quickly"* [Client 2]. Furthermore, the maternal healthcare clients save time and money when using the service and this motivate them to use CCPF. Instead of wasting transport money to visit the health centre just for advice, they just call CCPF at home while doing other thing at home.

Is there any information available on whether the women made these calls via other people's mobile phones / their own OR using landlines? Some clarity here on how they used the tele hotline would be helpful…

> *"We live far away from the health centre. Transport cost using a bicycle to the health centre is K1000 and to the district hospital is K1500"* [Client 3].

This finding shows that the mHealth intervention reduced the burden of walking long distances just to ask for maternal health information. In addition, the maternal healthcare clients saved financial resources that they may require simply to travel to access maternal health information. The maternal healthcare clients could invest this time instead in other productive ventures.

Privacy offered by the intervention

The mHealth intervention provided a private space for maternal healthcare clients and their husbands to ask sensitive questions that they could not ask at the antenatal clinic. Furthermore, the mHealth intervention motivated the husbands to participate in maternal-related issues.

> *"My husband was too shy to accompany me at the antenatal clinic. However, nowadays we call CCPF together, and ask questions that we could not ask in the presence of other people at the clinic"* [Client 8].

This shows that the hotline service of CCPF provided the maternal healthcare clients and their spouses a private space to discuss maternal-related issues with health professionals whilst at home. Most of

how did you establish this?

the maternal healthcare clients participated in this study had low literacy levels. This could mean that these maternal healthcare clients had knowledge deficiency on maternal-related information. Hence, they were motivated with the mHealth intervention.

Basic psychological needs

The findings suggest that the following psychological needs motivated maternal healthcare clients to use the mHealth intervention: community-enabled culture of sharing, frugal design of the mHealth intervention, and perceived value of the mHealth intervention.

Community-enabled culture of sharing

The maternal healthcare clients in this study were motivated to use the mHealth intervention due to the community-enabled culture of sharing practiced in their communities. The culture of sharing among communities in Balaka District created a feeling of connectedness, which is a basic psychological need. This finding was common among maternal healthcare clients who did not own mobile phones to access the mHealth intervention, or who had another mobile phone using another mobile provider other than Airtel Malawi. *"I used the mobile phone of a community volunteer to call CCPF or to listen to my messages ..."* [Client 4]. Furthermore, the maternal healthcare clients could use any mobile phone on the Airtel Malawi network to call or retrieve their messages for free.

Moreover, the community volunteers in Balaka District were visiting the maternal healthcare clients in their homes to tell them about CCPF mHealth intervention and the benefits of using the mHealth intervention. Community volunteers also assured maternal healthcare clients access of the project mobile phone for CCPF mHealth intervention. Hence, a supportive environment enforces a culture of sharing, which other mobile phone owners could imitate. This may promote an inclusive

culture. This could mean that communities of Balaka formed a CoP, which promoted an inclusive culture that could bring a sense of connectedness to the maternal healthcare clients. An inclusive culture has the potential to promote participation of maternal mHealth beneficiaries, which could have been potentially excluded in maternal mHealth interventions.

Frugal design of the mHealth intervention

The findings showed that the maternal healthcare clients were motivated to use the mHealth intervention since they found it easy to use.

> *"I can call CCPF by myself. I just call 54747 and choose Chichewa, then press 1 to talk to the doctor or 2 to listen to messages"* [Client 10].

By making the mHealth intervention easy to use, improved the technology-efficacy of the users of mHealth intervention. CCPF worked on a basic mobile phone. Furthermore, the intervention designed an easy-to-use method for the maternal healthcare clients to follow when they call the hotline toll-free number. In addition, the intervention is available in all the major local languages spoken in Malawi.

This finding suggests that the ergonomics of these information systems have the potential to motivate individuals to utilize them. Understanding the demographic characteristics of the potential beneficiaries, in terms of literacy and the type of mobile phone used could promote the design of intervention that can used on any mobile phone type, including basic mobile phones.

Perceived quality of information

Maternal healthcare clients in this study felt a sense of autonomy since the clients were able to call CCPF at anytime and anywhere. The sense of autonomy was enhanced by the maternal clients' perceived quality of information. Maternal healthcare clients in this study found the information they received from the mHealth intervention to be of good quality. The maternal healthcare clients

expressed that the information was relevant, since whatever advice they received worked for their specific problem. For example, the advice maternal healthcare clients receive from other women that eating eggs is not advisable for pregnant women. The maternal healthcare clients found that this is not true, since the messages from the mHealth intervention said that it is good to eat eggs while pregnant, because it is a significant source of protein supporting optimal pregnancy nutrition.

Other maternal healthcare clients found the information they received to be truthful. Perceived quality of information could be associated with the trust that maternal healthcare clients has with the provider of information. This trust motivated the maternal healthcare clients to choose CCPF as their preferred choice of source of information, which promoted maternal healthcare client autonomy.

Enablers to use mHealth interventions

A lot of the other aspects you have described above, e.g. perceived quality of information are also technically enablers… i suggest using a different name for this theme, e.g. access to, and attitudes towards technology…

Maternal healthcare clients were motivated to use maternal mHealth interventions due to enablers such as ownership or psychological ownership of the mobile phone and persuasion to use mHealth intervention.

Ownership or psychological ownership of the mobile phone

The study noted that maternal healthcare clients' ownership of a mobile phone was an enabler for them to use the intervention. Maternal healthcare clients who owned a mobile phone using an Airtel Malawi mobile line were motivated to use the mHealth intervention since they had the prerequisite technology that enabled them to access the intervention. By way of contrast, maternal healthcare clients whose mobile phones were with the other mobile operators, or who did not own a mobile phone, depended on a mobile phone that was not theirs in order to access the intervention. It could be the case that the maternal healthcare clients developed a sense of ownership over mobile

phones they did not own. This depended on the trust that the maternal healthcare client had with the mobile phone owner, and the relationship between the mobile phone owner and the maternal healthcare clients. The maternal healthcare clients used specific mobile phones for CCPF, and it was not common for the clients to use the phones of others for the intervention. This could be associated with pregnancy being considered a sensitive topic, with maternal healthcare clients using the mobile phones of those people whom they trust with their privacy. Hence, this shows that maternal healthcare clients used three types of mobile phone ownership: personal mobile phones, project mobile phones, and family mobile phones.

Personal mobile phones: The use of personal mobile phones was common among the maternal healthcare clients who owned mobile phones. Some community members who owned personal mobile phones could not share their mobile phones with maternal healthcare clients who did not own mobile phones, while others shared their personal mobile phone with maternal healthcare clients. *"For CCPF, I use the community member mobile phone… because this mobile phone is always available to me…"* [Client 15]. However, maternal healthcare clients commented that it was not easy to create a relationship and trust with any community member for them to be assured mobile phone usage for CCPF. This means that the maternal healthcare clients could not develop a feeling of possession on any mobile phone ,but depended on the agreement that they had with the mobile phone owner.

Project mobile phones: For mobile phones owned as a project mobile phone, maternal healthcare clients reported that the community volunteer mobile phone is a project-owned mobile phone. One of the maternal healthcare clients narrated that she knows that the community volunteer mobile phone is intended for everyone to use in their community. The findings of this study show that most of the maternal healthcare clients in the sampled area used project mobile phones.

Family mobile phone: Maternal healthcare clients who were using their husband's mobile phones felt that the mobile phone is a family mobile phone.

"I feel that the phone is a family phone but the husband is the main owner of the phone. I can use the phone when my husband is home and I cannot tell him that I want to use the phone when he is going to work, I have to wait for him to come home for me to use the phone" [Client 1].

The maternal healthcare clients showed accountability for the borrowed mobile phone by taking charge of the mobile phone when the maternal healthcare clients were using the mobile phone. *"I take care of the phone as my phone ..."* [Client 10].

Consider using different terminology here for the theme, e.g. persuasive approaches for promoting adoption

Persuasion to use mHealth intervention

The findings of this study show that maternal healthcare clients were persuaded to use mHealth intervention through different methods, such as awareness campaigns organized by the implementing agency; SMSs sent to all Airtel Malawi subscribers in the District; interpersonal communication; and posters. Through interpersonal communication, maternal healthcare clients in this study were persuaded to register for CCPF by other community members, HSAs, community volunteers, and their husbands. Maternal healthcare clients mentioned that they were persuaded to use CCPF mHealth intervention by HSAs and community volunteers, who visited them at their homes or met at social gatherings in their communities.

"I joined [CCPF] because of the advice I received from the HSA, that I can be helped while at home. Also, I can be listening to messages about my pregnancy... and when I deliver; I can also follow how my baby is growing" [Client 13].

For some clients, their husbands registered for them for the tips and reminders.

"My husband did the registration, so I do not know what they were talking about. However, when the messages come, we were reading them together, and I can call CCPF whenever I need help anytime ..." [Client 1].

This shows that persuasion to initially adopt an mHealth intervention was an enabler for the maternal healthcare clients to be motivated and continue using the mHealth intervention.

Internalization and integration

This entire section is more related to trust in information. I think this can be combined with the above theme. You can still comment on the growth of trust in the intervention with use. However, I don't think this theme has enough new information to be a standalone theme.

Even though internalization and integration of extrinsic motivation with basic psychological needs and enablers is not physical; we were able to observe the maternal healthcare clients before and after adopting the intervention. The maternal healthcare clients showed that after adopting the mHealth intervention they eventually trusted the information they received from the intervention. This means that the maternal healthcare clients were now intrinsically motivated to use the mHealth intervention. The maternal healthcare clients mentioned that they continued using the intervention even after they delivered their baby, up until the baby had received all the vaccinations.

Intrinsic motivation: trust in information received

The maternal healthcare clients in this study were motivated to use maternal mHealth intervention due to the trust in the information they received from the mHealth intervention. This was noted particularly among maternal healthcare clients with low literacy. The reason for this could be that the maternal healthcare clients did not have a chance to learn about some of pregnancy-related issues in school:

“CCPF information is very helpful, we were just staying and we were ignorant, we didn’t know what to do. Most women were dying because they did not know when to go to the hospital when problems arise, because of lack of proper advice …” [Client 17].

This finding shows that maternal healthcare clients developed trust in the information they were receiving from CCPF. This was also noted when maternal healthcare clients said that due to the quality of the information received from CCPF, *“whenever we are advised to visit the hospital by CCPF we go …”* [Client 17]. Trust in the information received from the mHealth intervention could have been emancipated since the maternal health information was provided by a government health professional. This finding shows that the trust in information received from the mHealth intervention enabled health facility usage by maternal healthcare clients for antenatal care, delivery, and postnatal care. Health facility usage for pregnancy-related issues is important to ensure improved maternal outcomes.

Discussion

The findings of this study suggest that maternal healthcare clients were motivated to use mHealth intervention due to the following reasons: 1) the technology suppressed social-cultural norms, 2) the affordance potency of the intervention, 3) trust in the source of information. And 4) the Ubuntu culture of sharing within communities.

This is the first time you are bringing up this terminology. I suggest providing the themes here in a similar language as you used above, e.g. inter-dependence. Then later in the discussion you can provide references and clarify this.

The technology suppressed social-cultural norms

The findings of this study suggest that the mHealth intervention suppressed social-cultural norms that would not allow pregnant women with socially unacceptable pregnancies to seek maternal-

related information and help or seek help from males and young CHWs and community members (17,25). The mHealth intervention enabled pregnant women whose pregnancies were not socially accepted to access necessary maternal healthcare information. These pregnant women could have been potentially excluded from benefiting from the mHealth intervention. Such a dilemma is common among unmarried teenage pregnant girls, who are normally shy to ask fellow women about maternal related information. Other studies have found that teenage girls and unmarried women who do own mobile phones could not use shared phones from infomediaries since their pregnancies were socially unacceptable (26). Others have stated that even when they attend antenatal clinics in person, they are not comfortable to ask questions (27).

In addition, the mHealth intervention also masked the gender and age of the service provider. This was the case since the maternal healthcare clients would not know whether the health worker was young, and when the health worker was male, due to the anonymity of the service. This enabled pregnant women to ask for maternal-related advice from hotline workers without the risk of conflicting with the social norms. This means that the mHealth intervention offers a *faceless* interaction, which assists in overcoming societal taboo (28). Moreover, faceless interaction with mHealth intervention assists in building trust with the intervention, which in turn promotes usage (29). Furthermore, faceless interactions provided the privacy that maternal healthcare clients and their husbands need to ask sensitive questions, which could have been shameful to ask at antenatal clinics due to social-cultural norms. For this reason, findings from this section suggest the following proposition:

Suggest re-wording - the tech is not suppressing norms, rather it is mitigating socio-cultural barriers

Proposition 1: *When technology suppresses social-cultural norms, maternal healthcare clients are motivated to use maternal mHealth interventions*

Designing interventions with affordance potency in mind

As before, you are introducing new information here without adding enough context from the actual findings. Perhaps move the findings about frugal design first.. and then in the context of that, introduce the new concept of affordance potency

Affordance potency refers to the strength of the relationship between the abilities of the individual and the features of the system at the time of actualization, conditioned by the characteristics of the environment (30). In this study, features of the CCPF, abilities of the maternal healthcare clients, and context of the maternal healthcare client strengthen the affordance potency.

The frugal design of the mHealth intervention made it easy to use. Maternal healthcare clients attested that CCPF was easy to use because it uses commonly used functions of a mobile phone. It could be the case that the maternal healthcare clients had the technical skills to use a basic mobile phone or the method used to access the intervention was simple. Hence, considering the context of use when designing mHealth interventions could motivate beneficiaries of the intervention to use them (Maliwichi et al., 2021b). Furthermore, considering the characteristics of the beneficiaries could influence mHealth designers to design interventions that match the capabilities of the users. For example, most of the maternal healthcare clients in this study had low literacy levels (17,32). For this reason, CCPF replaced SMS tips and reminders with pre-recorded IVR messages since most of the maternal healthcare clients could not read these messages (32). The frugal design of the intervention could promote *trialability* of the intervention, which in turn could promote adoption and use of the intervention (28,33). Hence, affordance potency has the potential to help in understanding the relationship between the abilities of users and system features (30).

Moreover, when the context where individuals reside is supportive, this may strengthen affordance potency. In this study, maternal healthcare clients had help from family members, community volunteers and other community members regarding how to use the service. This may

have strengthened the affordance potency. In addition, the use of community volunteers in communities instilled an inclusive culture in the communities and helped to shape user goals (34,35). For this reason, findings from this section suggest the following proposition:

Proposition 2: *When the design of the mHealth intervention is influenced by the affordance potency, maternal healthcare clients could be motivated to use the mHealth interventions.*

Trust in the source of information

Studies in maternal health have found that when health information is provided by the government, maternal healthcare clients are motivated to use the intervention because they trust in their government (36,37). This finding is similar to those in this study because the maternal healthcare clients knew that the Government of Malawi was involved since the intervention hotline workers were nurses and clinical officers working at a public hospital (Balaka District Hospital).

Studies in maternal mHealth have found that trusting beliefs in maternal mHealth influences initial acceptance and usage of maternal mHealth interventions (29). User beliefs in an mHealth intervention can influence its perceived efficacy (29,38). Hence, for maternal mHealth intervention to be successful, maternal healthcare clients need to believe in the information received from the mHealth intervention. In contrast, studies have also found that users tend not to be motivated to use technologies that they do not trust or are uncertain of their eligibility (38). Based on this discussion, the study suggests the following proposition:

Proposition 3: *When maternal healthcare clients trust the source of information, they are intrinsically motivated to use the mHealth interventions.*

Ubuntu culture of sharing within communities

In poor-resource settings, communities get resources through donor agencies, who brings resources in these communities such as food (soya flour and peanut butter) for children under the age of five in healthcare facilities (39). Mothers of under-five children take their children for immunization or attend antenatal clinics, expecting a gift for their attendance. The findings of this study suggest that the *culture of gifts* to coax maternal healthcare clients to use mHealth interventions could in this case have motivated the maternal healthcare clients to use the mHealth intervention. Other studies found

What does this mean? When introducing new information, please make sure to elaborate what you mean by this

that extrinsic compensation motivated university teacher's continuous intention to use flipped teaching (40). Furthermore, others found that compensation is one of the drives that motivate people (41). For this reason, the expectation to be compensated may motivate beneficiaries of interventions to use them.

Other studies have found that the *culture of gifts* is problematic, especially when the donor withdraws from the community (39). This could de-motivate certain beneficiaries. However, the fact that these beneficiaries had a chance to adopt the intervention has a chance to experience the benefits of the interventions and could eventually be intrinsically motivated to use the intervention. For this reason, it is beneficial for implementers of interventions to practice the culture of gifting, which promotes the initial adoption of interventions.

Moreover, when communities practice the *ubuntu* culture of sharing, for example, in the sharing of mobile phones with other beneficiaries who do not own mobile phones; it motivates these beneficiaries to use mHealth interventions. An *Ubuntu* culture of sharing has the potential to promote the inclusion of beneficiaries who may have been potentially excluded. This in turn promotes inclusive participation of mHealth intervention for all mHealth beneficiaries. The findings from this section suggest the following propositions:

Proposition 4a: *When communities practice the ubuntu culture of sharing, maternal healthcare clients who lack prerequisite technologies may be motivated to use interventions.*

Proposition 4b: *When implementers of mHealth practice a culture of gifting, maternal healthcare clients may be motivated to adopt and use the intervention.*

Conclusion and recommendations

When pregnant women are challenged with long distances to health facilities for health information and advice, mHealth interventions have the potential to bridge the gap. Women in rural areas might be motivated to use mHealth interventions when the technology suppresses social-cultural norms, and the technology is designed with affordance potency in mind, when the women have trust in the source of information, and when communities practice the *ubuntu* culture of sharing.

We therefore recommend that mHealth intervention designed for resource-constrained settings ought to be frugal. Most of the beneficiaries in these settings could use basic mobile phones and could have low levels of literacy. When mHealth designers take into consideration the characteristics of the beneficiaries, they may be able to tailor their interventions to context, thereby motivating potential beneficiaries to use the interventions.

The study also noted that the source of information matters when it comes to health information. Therefore, mHealth implementers ought to align themselves with the government health system for the potential beneficiaries to trust the health information. Moreover, government support would make the scaling-up of the intervention easy, thereby reaching more beneficiaries.

We also recommend implementers of interventions to identify the systems of sharing in communities and encourage them. These systems of sharing could promote an inclusive culture in communities to ensure that those beneficiaries who could be potentially excluded use the intervention. An inclusive culture promotes connectedness, which is a basic psychological need. This in turn promotes the culture of sharing in communities that can assist the less fortunate within communities to enjoy access to prerequisite technologies to use interventions that might fundamentally alter their quality of life. In addition, we recommend that mHealth implementers use extrinsic motivation to promote the trialability of the technology. This could bring in beneficiaries to try the intervention which eventually could lead to intrinsic motivation, which promotes the adoption and use of interventions.

The study was based in only one district in Malawi, and we only interviewed 20 participants. This may not be enough to generalize the results to all mHealth interventions in the country. Therefore, future work could test the extended framework in similar settings.

Acknowledgments

None

References

1. WHO. World Health Statistics 2018 - Monitoring health for the SDGs, sustainable development goals. 2018. p. 1–100.

2. Blauvelt C, West M, Maxim L, Kasiya A, Dambula I, Kachila U, et al. Scaling up a health and nutrition hotline in Malawi: The benefits of multisectoral collaboration. BMJ. 2018;363:k4590.

3. Watkins SC, Robinson A, Dalious M. Evaluation of the Information and Communications Technology for Maternal, Newborn and Child Health Project [Internet]. 2013. Available from:

http://www.villagereach.org/wp-content/uploads/2017/07/ICT_for_MNCH_Report_131211md_FINAL.pdf

4. World Health Organization. World Health Statistics 2018 - Monitoring health for the SDGs, sustainable development goals [Internet]. 2018. p. 1–100. Available from: https://www.who.int/gho/publications/world_health_statistics/2018/en/

5. Lund S, Hemed M, Nielsen BB, Said A, Said K, Makungu MH, et al. Mobile phones as a health communication tool to improve skilled attendance at delivery in Zanzibar: a cluster-randomised controlled trial. International Journal of Obstetrics & Gynaecology. 2012;119(10):1256–64.

6. Kemp S. Datareportal. 2020. Digital 2020: Malawi. Available from: https://datareportal.com/reports/digital-2020-malawi

7. Maliwichi P, Mthoko H, Chigona W, Mburu C, Densmore M. Does mobile phone ownership matter? Insights on engagement in mHealth and e-government interventions from Southern Africa. Int J Health Promot Educ [Internet]. 2021;1–22. Available from: https://doi.org/10.1080/14635240.2021.1995775

8. Global System for Mobile Communications Association [GSMA]. The Mobile Gender Gap report 2019 [Internet]. 2019. Available from: https://www.gsma.com/mobilefordevelopment/resources/mobile-gender-gap-report-2019/

9. VillageReach. Evaluation of the Information and Communications Technology for Maternal Newborn and Child Health Project (Chipatala cha pa Foni) [Internet]. 2014. Available from: https://www.villagereach.org/wp-content/uploads/2009/08/VR-Exec-Summary-of-CCPF-Eval_Feb-21_FINAL.pdf

10. Thohir L. Motivation in a Foreign Language Teaching and Learning. JOURNAL FOR LANGUAGE AND FOREIGN LANGUAGE LEARNING. 2017;6(1):20–8.

11. Medhanyie AA, Little A, Yebyo H, Spigt M, Tadesse K, Blanco R, et al. Health workers' experiences, barriers, preferences and motivating factors in using mHealth forms in Ethiopia. Hum Resour Health. 2015;13:2.

12. Modi D, Desai S, Dave K, Shah S, Desai G, Dholakia N, et al. Cluster randomized trial of a mHealth intervention "ImTeCHO" to improve delivery of proven maternal, neonatal, and child care interventions through community-based Accredited Social Health Activists (ASHAs) by enhancing their motivation and strengthening. BioMed Central. 2017;18:270.

13. Bedrov A, Bulaj G. Improving self-esteem with motivational quotes: Opportunities for digital health technologies for people with chronic disorders. Front Psychol. 2018 Nov 2;9(OCT).

14. Ba S, Wang L. Digital health communities: The effect of their motivation mechanisms. Decis Support Syst. 2013 Nov;55(4):941–7.

15. Zinsser LA, Stoll K, Wieber F, Pehlke-Milde J, Gross MM. Changing behaviour in pregnant women: A scoping review. Midwifery. 2020;85:102680.

16. Guendelman S, Broderick A, Mlo H, Gemmill A, Lindeman D. Listening to communities: Mixed-method study of the engagement of disadvantaged mothers and pregnant women with digital health technologies. J Med Internet Res. 2017;19(7).

17. Larsen-Cooper E, Bancroft E, Toole MO, Jezman Z. Where there is no phone: The benefits and limitations of using intermediaries to extend the reach of mHealth to individuals without personal phones in Malawi. African Population Studies Special Edition. 2015;29(1):1628–42.

18. Ryan RM, Deci EL. Intrinsic and extrinsic motivation from a self-determination theory perspective: Definitions, theory, practices, and future directions. Contemp Educ Psychol. 2020;61:101860.

19. Visser CLF, Kusurkar RA, Croiset G, ten Cate O, Westerveld HE. Students' motivation for interprofessional collaboration after their experience on an IPE ward: A qualitative analysis framed by self-determination theory. Med Teach. 2019;41(1):44–52.

20. Creswell JW. Research Design - Qualitative, Quantitative and Mixed Methods Approaches. 4th ed. Thousand Oaks, CA: Sage Publication; 2014. 413 p.

21. Mobile Alliance for Maternal Action (MAMA). Chipatala Cha Pa Foni. Sportlight. 2013;(September):1–9.

22. National Statistical Office [NSO] and ICF Macro. Malawi DHS, 2010 [Internet]. NSO and ICF Macro. Zomba, Malawi, and Calverton, Maryland, USA; 2011. Available from: https://tbinternet.ohchr.org/Treaties/CEDAW/Shared Documents/MWI/INT_CEDAW_ADR_MWI_19521_E.pdf

23. VillageReach. Press Release:National expansion plan for Chipatala Cha Pa Foni (CCPF): A health and nutrition hotline [Internet]. 2018. Available from: http://www.villagereach.org/press-release-national-expansion-plan-chipatala-cha-pa-foni-ccpf-health-nutrition-hotline/

24. Braun V, Clarke V. Using thematic analysis in psychology. Qual Res Psychol. 2006;3(2):77–101.

25. Duclos V, Yé M, Moubassira K, Sanou H, Sawadogo NH, Bibeau G, et al. Situating mobile health: A qualitative study of mHealth expectations in the rural health district of Nouna, Burkina Faso. Health Res Policy Syst. 2017;15(Suppl 1).

26. Maliwichi P, Chigona W. Factors affecting the use of infomediaries in mHealth interventions: Case of maternal healthcare in rural Malawi. In: NEMISA Summit and Colloquium 2022. 2022. p. 1–30.

27. Hackett K, Lenters L, Vandermorris A, Lafleur C, Newton S, Ndeki S, et al. How can engagement of adolescents in antenatal care be enhanced? Learning from the perspectives of young mothers in Ghana and Tanzania. BMC Pregnancy Childbirth. 2019;19:184.

28. Sowon K, Maliwichi P, Chigona W. The Influence of Design and Implementation Characteristics on the Use of Maternal Mobile Health Interventions in Kenya: Systematic Literature Review. JMIR Mhealth Uhealth. 2022;10(1):e22093.

29. Sowon K, Chigona W. Trust in mHealth: How do Maternal Health Clients Accept and Use mHealth Interventions? ACM International Conference Proceeding Series (2020). 2020;189–97.

30. Anderson C, Robey D. Affordance potency: Explaining the actualisation of technology affordances. Information and Organization. 2017;27(April):100–15.

31. Maliwichi P, Chigona W, Sowon K. Appropriation of mHealth Interventions for Maternal Healthcare in sub-Saharan Africa: Hermeneutic Review. JMIR Mhealth Uhealth. 2021;9(10):e22653.

32. Crawford J, Larsen-Cooper E, Jezman Z, Cunningham SC, Bancroft E. SMS versus voice messaging to deliver MNCH communication in rural Malawi: Assessment of delivery success and user experience. Glob Health Sci Pract. 2014;2(1):35–46.

33. Rogers EverettM. Diffusion of innovations. 5th ed. New York: Free Press; 2003.

34. Mohd NMA, Mamat MN, Mahamood SF, Mohd NMM. ASEAN as an Inclusive Community: Identifying and Overcoming the Challenges. In: Kaur N, Ahmad M, editors. Charting a Sustainable Future of ASEAN in Business and Social Sciences. Singapore: Springer; 2020. p. 215–24.

35. Zamani ED, Sbaffi L. Embracing uMunthu: How Informal Caregivers in Malawi Use ICTs. In: Bass JM, Wall PJ, editors. Information and Communication Technologies for Development ICT4D 2020 IFIP Advances in Information and Communication Technology, vol 587. Springer, Cham; 2020. p. 93–101.

36. Seebregts C, Dane P, Parsons AN, Fogwill T, Rogers D, Bekker M, et al. Designing for scale: Optimising the health information system architecture for mobile maternal health messaging in South Africa (MomConnect). BMJ Glob Health. 2018;3:e000563.

37. Grameen Foundation. Mobile Technology for Community Health in Ghana:What it is and what Grameen Foundation has learned so far [Internet]. Washington D.C: Grameen Foundation; 2012. 148 p. Available from: https://courses.cs.washington.edu/courses/cse490d/12sp/docs/MOTECH.pdf

38. Akter S, D'Ambra J, Ray P. Trustworthiness in mHealth information services: An assessment of a hierarchical model with mediating and moderating effects using partial least squares (PLS). Journal of the American Society for Information Science and Technology. 2011;62(1):100–16.

39. Park JD. Assessing the Role of Foreign Aid, Donors and Recipients. In: Re-Inventing Africa's Development. Palgrave Macmillan, Cham; 2019. p. 37–60.

40. Lai HM, Hsiao YL, Hsieh PJ. The role of motivation, ability, and opportunity in university teachers' continuance use intention for flipped teaching. Comput Educ. 2018;124(2018):37–50.

41. Muguongo MM. Effects of Compensation on Job Satisfaction Among Secondary School Teachers in Maara Sub - County of Tharaka Nithi County, Kenya. Journal of Human Resource Management. 2015;3(6):47–59.

42. Jimat Development Consultants. Impact Evaluation of Chipatala cha pa Foni (CCPF), Malawi's Health and Nutrition Hotline [Internet]. 2018. Available from: https://www.villagereach.org/wp-content/uploads/2020/02/VR_CCPFImpactEval_FINAL.-2_24_20.pdf

43. JIMAT Development Consultants. Impact Evaluation of Malawi's Maternal Child Health and Nutrition Hotline: Chipatala Cha Pa Foni (CCPF). 2018.

44. Innovation Working Group (IWG). CHIPATALA CHA PA FONI: Healthcare Through Mobile Phones. 2016.

45. Fotso JC, Bellhouse L, Vesel L, Jezman Z. Strengthening the home-to-facility continuum of newborn and child health care through mHealth : Evidence from an intervention in rural Malawi. African Population Studies Special Edition. 2015;29(1):1663–83.

Supporting Information

SI Table 1. *Summary of secondary data sources*

Document title	Document Type	References
Impact evaluation for CCPF, Malawi’s health and nutrition hotline - Full proposal	Project Information	(42)
Impact Evaluation of Malawi’s Maternal Child Health and Nutrition Hotline: Chipatala Cha Pa Foni (CCPF) - Preliminary Report	Project information and CCPF usage	(43)
Chipatala Cha Pa Foni: Healthcare through mobile phone	Project Information	(44)
Evaluation of the Information and Communication Technology for Maternal, Newborn and Child Health Project: Improving Access to Reproduction, Maternal and Newborn Health Information and Services in Malawi	CCPF usage Report	(3)
Where there is no phone: The benefits and limitations of using intermediaries to extend the reach of mHealth to individuals without personal phones in Malawi	Published article	(17)
Scaling up a health and nutrition hotline in Malawi: The	Published article	(2)

benefits of multi-sectoral collaboration		
SMS versus voice messaging to deliver MNCH communication in rural Malawi: Assessment of delivery success and user experience	Published article	(32)
Strengthening the home-to-facility continuum of newborn and child health care through mHealth : Evidence from an intervention in rural Malawi	Published article	(45)

SI Table 2. *Demographic characteristics of the maternal healthcare clients*

**Characteristic**	**No of Clients**
Age range: 15-19	2
20-25	6
26-35	8
36-45	4
**Educational level**	
Primary	17
Junior Secondary	3
**No. of pregnancy used CCPF**	
1	13
2	4
3	3
**Months started ANC**	7
2	13
3	
**No of ANC visits**	
4	9
5	11

Click here to access/download
Related Manuscript
Extrinsic Motivation-Published.pdf
