# Supplementary material for: Factors motivating maternal healthcare clients to use mHealth interventions in rural Malawi
Source: PLOS Digit Health. 2025 Apr 22;4(4):e0000805. doi: 10.1371/journal.pdig.0000805 (PMC12013878; doi:10.1371/journal.pdig.0000805)
Supplement: Attachment — Submitted filename: Reviewers and editors comments responses.docx [file pdig.0000805.s006.docx]

**Reviewers and editors comments responses**

|  | **Editors comments** | **Responses** |
| --- | --- | --- |
| 1 | **Abstract** highlighted text | This text was deleted |
|  | **Introduction** |  |
| 2 | Line 45 – 46, the two highlighted sentences | These two sentence were deleted |
| 3 | Line 49 – Highlighted sentence | This sentence was moved to the next parahraph which now introduces the CCPF mHealth intervention |
| 4 | Line 52 – Hightlighted text | This was move to the paragraph whci introduced CCPF – and it was revised as advised in the comment in pink |
| 5 | Line 65 – highlighted text | The research question was deleted. But was incorporated in the revised last paragraph |
| 6 | Line 67 – highlighted text | This sentence was put in the second paragraph which introduces the case study. This author feels that it is important for the leader to understand what the phrase Chipatala Cha Pa Foni means. |
| 7 | Line 75 - highlighted text | This sentence was deleted |
|  | **Material** and Methods |  |
| 8 | Line 172 - Please provide interview questions as supplementary materials, and include examples of questions asked in the paper (If possible, please provide English translation of interview questions). | Line 183 – highlights area of interview questions. The specific questions were added in the supplementary materials |
| 9 | Line 178 - Please unpack the SDT further - what does it say? what are the components of self-determination, etc. according to this theory? | Line 193 – 210 introduce SDT |
| 10 | Line 185 - Please provide ethics approval / project ID if possible | Ethics approval protocol number has been provided |
| 11 | Line 189 - Was written consent sought? Or verbal? If verbal, was consent recorded? Also what are the challenges of informed consent? Did your participants have adequate literacy levels to understand the project? how did you ensure that the consent they were giving was informed consent? | We have revised the paragraph to address these concerns in lines 228 – 231. |
| 12 | Line 190 - Women who are no longer pregnant but had difficulties during pregnancy or childbirth could also | We have added how we mitigated this risk. Line 234 |
| 13 | Line 191- Highlighted text | This was deleted |
| 14 | Line 220 – Highlighted text | This sentence was deleted |
| 15 | Line 222 – highlighted word | This was edited |
| 16 | Line 228 – Highlighted phrase | Has been revised as suggested |
| 17 | Line 237- what are the other reasons for this? Is it just the above that you have described? | Yes, it is just what we have described. |
| 18 | Line 247 - Is there any information available on whether the women made these calls via other people’s mobile phones / their own OR using landlines? Some clarity here on how they used the tele hotline would be helpful… | We added this information on line 292 |
| 19 | Line 263 - how did you establish this? | Line 309, clarifies this concern by adding some information |
| 20 | Line 291 -296 – Highlighted text | This was deleted |
| 21 | Line 311 – 315 - A lot of the other aspects you have described above, e.g. perceived quality of information are also technically enablers… i suggest using a different name for this theme, e.g. access to, and attitudes towards technology… | Changed the theme as advised and moved the sub-theme perceived quality of information to this theme |
| 22 | Line 158 - Consider using different terminology here for the theme, e.g. persuasive approaches for promoting adoption | Changed the sub-theme as advised |
| 23 | Line 377 - This entire section is more related to trust in information. I think this can be combined with the above theme. You can still comment on the growth of trust in the intervention with use. However, I don’t think this theme has enough new information to be a standalone theme. | The theme Internalizaton and integrations was removed and we have only trust in information and in the intervention. |
| 24 | Line 407 - This is the first time you are bringing up this terminology. I suggest providing the themes here in a similar language as you used above, e.g. inter-dependence. Then later in the discussion you can provide references and clarify this. | Changed the theme as advised and updated the section. |
| 25 | Line 430 - Suggest re-wording - the tech is not suppressing norms, rather it is mitigating | Revised as advised. |
| 26 | Line 432 - As before, you are introducing new information here without adding enough context from the actual findings. Perhaps move the findings about frugal design first.. and then in the context of that, introduce the new concept of affordance potency | Revised the section as advised |
| 27 | Line 480 - What does this mean? When introducing new information, please make sure to elaborate | This sentence has been removed |
| 28 | Line 506 – Highlighted text | Was removed |
|  |  |  |
|  | **Reviewer 1 comments** | **Responses** |
| 29 | There are spelling issues (e.g., line 222, you might have meant realizing instead of releasing) | This was revised |
| 30 | sentences that did not flow well (e.g., line 293-294). Please go through the paper again paying attention to these details. | The sentences were deleted and the section was revised. |
| 31 | Great work in setting the scene in the introduction on the need and for choosing a very relevant case study. | This is noted |
| 32 | In the limitation section, you highlighted that there were only 20 people which limits the generalizability of the findings. There is a potential to expand more on the demographic of the participants in table 2 to provide a richer context of the findings in the discussion. | We have added information on mobile phone ownership |
| 33 | It is unclear if they received renumeration by participating in the interview, and whether their freedom in sharing their full experience was emphasized. | Yes the participants were compensated for their time . We have revised the ethics section. |
| 34 | It would also be good to include more detailed information of the mean and range of when they previously used the system, and similar reflections on their interaction with the system to provide context of use. | In this regard, we only collected the number of pregnancies one used the intervention. |
|  |  |  |
|  | **Reviewer 2 comments** | **Responses** |
| 35 | Theoretical Framing of Trust: Further elaboration on the theoretical underpinnings of trust in mHealth would strengthen the analysis. Exploring different dimensions of trust (e.g., trust in information sources, technology, and systems) could provide a more nuanced understanding. | The theoretical framing of trust was revised as advised. |
| 36 | Sustainability Beyond Extrinsic Motivation: While acknowledging the limitations of extrinsic motivators like mosquito nets, a deeper exploration of strategies for cultivating sustained engagement and integrating mHealth within existing healthcare systems would enhance the practical implications. | This has been factored in. |
| 37 | Generalizability and Future Research: The authors acknowledge the limitations of a single case study. Future research could explore the transferability of these findings to different contexts, considering cultural variations, technological infrastructure, and health system structures. | This has been added in the future work. |
|  |  |  |
